# E-Cadherin gene expression in oral cancer: Clinical and prospective data

**DOI:** 10.4317/medoral.23029

**Published:** 2019-07

**Authors:** Sandra López-Verdín, Margarita de la Luz Martínez-Fierro, Idalia Garza-Veloz, Ana Zamora-Perez, Jonathan Grajeda-Cruz, Rogelio González-González, Nelly Molina-Frechero, Miguel Arocena, Ronell Bologna-Molina

**Affiliations:** 1 Health Sciences University Center, Instituto de Investigación en Odontología, Universidad de Guadalajara, Jalisco, México; 2 Academic Unit of Human Medicine and Health, Universidad Autónoma de Zacatecas, Zacatecas, México; 3 Department of Research, School of Dentistry, Universidad Juárez del Estado de Durango, Durango, México; 4 Health Care Department, Universidad Autónoma Metropolitana, Mexico City, Mexico; 5 Biochemistry, School of Dentistry, Universidad de la República, Montevideo, Uruguay; 6 Molecular Pathology area, School of Dentistry, Universidad de la Republica, Montevideo, Uruguay

## Abstract

**Background:**

Low protein expression of E-cadherin in oral squamous cell carcinoma (OSCC) has been associated with clinical and histopathological traits such as metastases, recurrence, low survival and poor tumor differentiation, and it is considered a high-risk marker of malignancy. However, it is still unknown whether low expression of E-cadherin is also present at the mRNA level in OSCC cases. Objective: The aim of this study was to compare E-cadherin mRNA expression in OSCC patients and controls and to correlate the expression with clinical and prospective characteristics.

**Material and Methods:**

Forty patients and 40 controls were enrolled. E-cadherin mRNA expression was evaluated by quantitative real-time polymerase chain reaction using TaqMan probes.

**Results:**

E-cadherin mRNA expression was significantly decreased in OSCC patients compared to that of controls (*p*<0.001). Whereas no significant association between clinical parameters and E-cadherin expression levels was observed, we noted lower E-cadherin expression levels in patients with positive lymph node metastasis.

**Conclusions:**

E-cadherin mRNA expression was markedly diminished in OSCC, in agreement with previous results that examined E-cadherin expression at the protein level. E-cadherin is downregulated in the early clinical stages of OSCC, and its mRNA levels do not change significantly in the advanced stages, suggesting that there is limited usefulness of this parameter for predicting disease progression.

** Key words:**Oral cancer, E-Cadherin, gene expression.

## Introduction

Global demographic transitions suggest that there will be an increase in cancer burden over the next several decades, particularly in middle- and low-income countries (1). In 2012, GLOBOCAN reported that oral cancer made up approximately 2.1% of newly registered cancer cases. According to this study, approximately 145,000 deaths occurred because of oral cancer 77% of which occurred in less developed regions (2).

Oral squamous cell carcinoma (OSCC) is the most common histological cancer type of the oral cavity (3), and it is considered to have a multifactorial etiology due to the synergy of multiple risk factors (4,5).

In the advanced stages, neoplastic cells undergo a dedifferentiation process, which is followed by a loss of intercellular adherence. Intercellular adhesion is primarily maintained by cadherin adhesion molecules, and specifically by E-cadherin in the epithelial tissue (6). E-cadherin is a 120 kDa transmembrane glycoprotein encoded by the CDH1/E-cadherin gene located on chromosome 16q22.1, and it is considered a tumor suppressor gene due to its ability to negatively regulate cell proliferation (7). E-cadherin downregulation normally occurs during embryogenesis in the context of an epithelial-mesenchymal transition (EMT) (8-10), which allows cells to migrate to other embryonic layers. Similarly, neoplastic cells migrate and reach distant organs in malignant tumors of epithelial origin after undergoing an EMT that involves decreased E-cadherin expression (8).

Accordingly, low expression of E-cadherin in OSCC has been associated with clinical and histopathological features of malignancy, such as metastasis, recurrence, low survival and poor tumor differentiation (11-13), and it has been referred to by some authors as a “high-risk marker of malignancy” (14,15). However, in most studies, E-cadherin expression has been assessed at the protein level using immunohistochemistry (12,15-23). Cancer is a genomic disease (24), and gene expression at the mRNA level of markers involved in carcinogenesis, such as E-cadherin, needs to be evaluated in OSCC in the context of clinical characteristics associated with the disease. The aim of this study was to quantify the gene expression levels of E-cadherin in oral cancer patients and controls and to evaluate their association with tumor, node, metastasis (TNM) stage, persistence, recurrence, death, and response to therapy.

## Material and Methods

A total of 40 cases of primary OSCC were diagnosed over a period of 3 years (2012-2013) (24 males and 16 females, aged 60.8 ± 15.7 years) at the Instituto Jaliscience de Cancerología (Jaliscience Cancer Institute); no anticancer therapy had been initiated in any of these cases. The 40 control samples were obtained from patients (22 male and 18 female subjects aged 58.7 ± 17.5 years) who visited the maxillofacial surgery service at the Hospital Civil Nuevo (New Civil Hospital) for tooth extraction with prosthetic and orthodontic indications. For each patient, data regarding the following clinico-pathological factors were collected: gender, age, smoker status, alcohol consumption, TNM stage, recurrence, persistence and date of death. The exclusion criteria for both groups were having or having had any type of cancer other than OSCC, being diagnosed with an autoimmune disease that affected the oral cavity, such as pemphigus vulgaris or lichen planus, or not signing the informed consent. Protocol was reviewed and accepted by Universidad de Guadalajara biosecurity and etic committee number CI-02112

-Clinical and prospective data

The stage of the disease was evaluated at the Surgical Head and Neck Oncology Department of the Jaliscience Cancer Institute, and each tumor was analyzed clinically and by computerized tomography. The disease stages were then determined according to the TNM system (AJCC) (25).

Due to the finding that the diameter of the tumor and invasion into lymphatic nodes are regarded as important clinical markers involved in prognosis, the lymphatic nodes were considered positive if patients presented with metastasis in one or more lymphatic nodes. T stage and total TNM scores were assigned in accordance with the National Cancer Institute guidelines, and each case was determined to be early- or advanced-stage disease (NCI, 2016) (26). According to this system, advanced T stage refers to tumors that are larger than 4 cm, whereas advanced TNM stage also incorporates tumors of a smaller size that are accompanied by positive lymphatic nodes with or without distant metastasis.

Prospective data were taken and updated each year after diagnosis and at the beginning of anticancer treatment. The average follow-up was 43.02 ± 13.48 months, with a minimum of 21 months and a maximum of 65 months. Persistence was diagnosed in patients who showed tumoral activity based on imaging studies after a 9 to 12 month period after the conclusion of therapy, according to the criteria previously described by DeSouza *et al.* (27). Additionally, recurrence of the disease was defined as, despite there being a period of time with eradication of clinical and imaging manifestations of disease, also known as disease-free period or disease-free interval, the reappearance of cancer, whether in the location of the original tumor (local recurrence), in regional lymph nodes or in adjacent tissues (regional recurrence), or if it had disseminated to any other body part (distant recurrence) (28). A patient was considered to be a non-responder to anticancer therapy if they experienced recurrence and/or persistence.

-Sample retrieval and storage

A fresh tissue sample that was at least 5 mm in diameter was obtained at the time of the surgical procedure, during biopsy in the OSCC group and tooth extraction in the control group. Samples were stored in a -80°C freezer.

-RNA isolation and cDNA synthesis

RNA extraction from tissue samples was performed using TRIzol (Invitrogen®) reagent coupled with Polytron® (Kinematica Inc.) homogenization at room temperature (RT). Afterwards, 200 µl of chloroform was added for every 750 µl of TRIZOL. Samples were shaken vigorously using a vortex for 15 seconds, incubated at room temperature for 5 minutes, and centrifuged at 11000 rpm for 10 minutes at 4°C. The upper aqueous phase was transferred carefully to precipitate the RNA by mixing with 1 volume of isopropanol. Samples were incubated at RT for 15 minutes and centrifuged at 14000 rpm for 10 minutes at 4°C. Next, samples were washed with 80% ethanol in diethyl pyrocarbonate water, and the RNA pellet was air-dried for 5-10 minutes. RNA concentration and quality were measured using a NanoDrop Spectrophotometer (NanoDrop Technologies, Wilmington, DE). cDNA was synthesized from 100 ng of total RNA using a High Capacity cDNA Reverse Transcription Kit (Applied Biosystems) according to the manufacturer’s instructions. cDNA samples were stored at −20°C.

-Relative quantification by Real-Time Polymerase Chain Reaction (qRT-PCR)

Before quantification of the gene of interest, selection of an endogenous gene for normalization was performed by construction of standard curves. In this stage, a total of 100 ng cDNA was taken from 6 OSCC cases and 6 controls to verify that the endogenous gene had no variation between study groups. Five serial dilutions of each cDNA were made to generate a concentration range from 25 ng to 200 ng. The qRT-PCR reactions to evaluate the 18S RNAr, B2M, GAPDH and HPRT1 endogenous genes were performed in a final volume of 10 µL, 1X TaqMan Universal PCR Master mix, 1X TaqMan Gene Expression Assay primer and probe mix (Applied Biosystems). The qPCR reactions were performed on a StepOne Plus Real-Time PCR System (Applied Biosystems, Foster City, CA) according to the manufacturer´s instructions. Standard curves were compared using Student’s t-test. The gene 18S RNAr was selected because it had the lowest significance.

The relative mRNA expression levels of E-cadherin were determined using a TaqMan® Gene Expression Assay (Hs01023894_m1; Applied Biosystems). The TaqMan probe corresponded to the cdh1/E-cadherin gene joining at exons 2 and 3 from the 284 bp position and amplified a fragment of 61 bp. During the quantification of E-cadherin gene expression using the qRT-PCR assay, 20 ng of cDNA from each patient sample was used in duplicate for each PCR (according to the mixing conditions previously described). The Cq values were normalized to those of the 18S-rRNA endogenous gene, and gene expression was calculated as the fold-change relative to the average expression of the controls using the 2-ΔΔCt method.

-Immunohistochemistry (IHC)

To confirm the protein presence in the tumoral tissue, immunohistochemical technique was performed. . Tissue samples were processed and evaluated. Three-μm-thick sections were cut, placed on poly-L-lysine-coated slides, deparaffinized in a 60°C oven for 30 minutes and incubated in xylol for 5 minutes. The sections were then rehydrated in decreasing alcohol concentrations (absolute, 90%, 70%, and 50%) and washed in distilled water. To retrieve the epitopes, the tissue sections were incubated in 10 mM sodium citrate solution, pH 9, in a microwave oven at a maximum power of 750 W for two cycles of 5 minutes each. The sections were then cooled to room temperature and washed with distilled water. The endogenous peroxidases were blocked with 0.9% hydrogen peroxide, and the samples were washed again with distilled water and phosphate-buffered saline solution (PBS, pH 7.4). The sections were incubated for 30 minutes with primary antibodies against E-cadherin (Clone NCH-38, 1:50, Dako Corp., Carpinteria, CA, USA). Next, the sections were incubated with a biotinylated anti-mouse/anti-rabbit secondary antibody and a streptavidin/peroxidase complex (LSAB+-labeled streptavidin-biotin, Dako Corp.) for 30 minutes each, and the reaction products were detected with 3,3′-diaminobenzidine-H2O2 (Dako Corp.).

For microarray analysis, negative and positive controls were obtained from paraffin blocks containing samples of healthy mucosa and fibroma using a 5-mm punch and were placed on slides along with the samples.

The immunopositivity and immunolocalization were analyzed by an oral pathologist. The E-cadherin membranal protein (mP) was expressed on the cell membrane in all tumor tissues, regardless of the high or low expression status.

-Statistical Analysis

Comparisons were made between OSCC cases and controls by Student’s t-test. To perform Chi-square or Fisher’s exact test, the conversion of categorical variables was necessary; zero was considered to be the cutoff point for under- or overexpression. Comparisons between mRNA E-cadherin expression levels, mP E-cadherin and disease characteristics, such as TNM and TNM clinical stage, and prospective data were calculated by the Mann-Whitney U-test and Chi square. Statistical analysis was performed using SPSS statistics v23 software, and ≤ 0.05 was considered to be statistically significant.

## Results

Sociodemographic and risk factor profiles from the OSCC case and control groups were obtained. Both groups were similar in regard to gender and age distribution as well as smoking status, whereas alcohol consumption was higher in the control group. With the purpose of assessing the influence of risk factors on E-cadherin gene expression, a comparative analysis between E-cadherin mRNA levels and alcohol or tobacco consumption was made in both case and control groups, without showing significant differences (t-student test, *p*-values are shown in  Table 1).

**Table 1:**
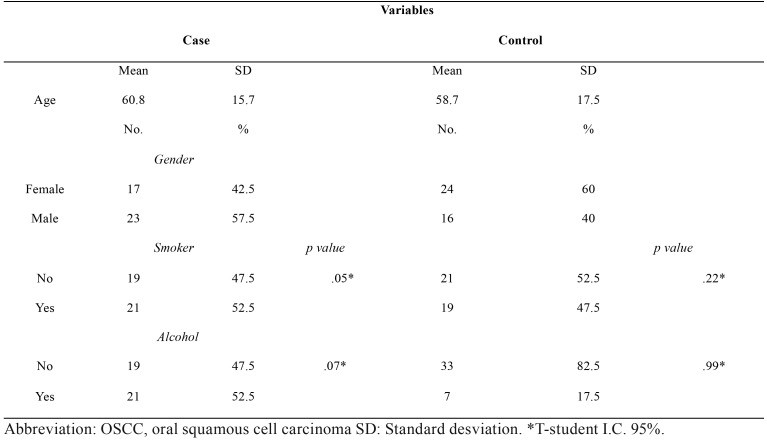
Sociodemographic, risk factor descriptive data and expression mRNA E-cadherin difference from OSCC patients and the control group.

The description of the clinical characteristics and prospective data of patients with oral cancer showed that a majority of the patients presented tumors larger than 4 cm, with regional lymph node metastasis and advanced TNM clinical stage ( Table 2). However, none of the patients showed dissemination to distant organs. From the prospective data, it should be highlighted that most of the patients responded to the therapy and were still alive at the time that this paper was completed.

**Table 2:**
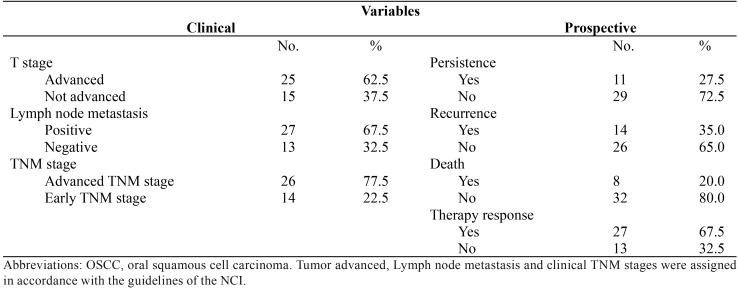
Clinical and prospective data from 40 OSCC patients.

**Figure 1:**
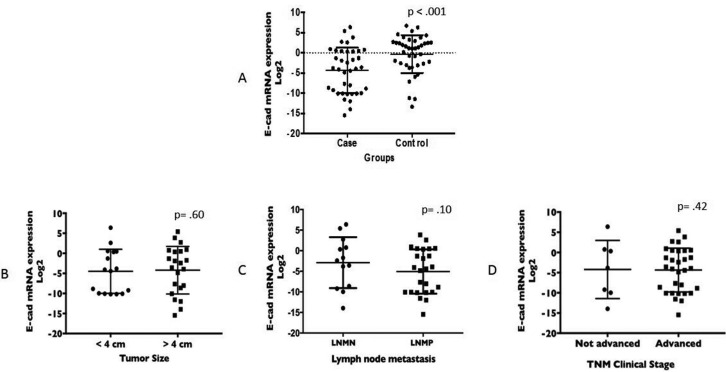
Student’s t-test. A) Comparison of the E-cadherin gene expression values between the OSCC case (mean -4.32 ± 5.67; minimum -15.46; maximum 6.38) and control (mean -.31 ± 4.72; minimum -13.32; maximum 6.70) groups. Mann–Whitney test. Comparison of the E-cadherin gene expression values in the T, N and TNM clinical stages. B) Tumor not advanced (< 4 cm) vs Tumor advanced (> 4 cm): mean 4.76 ± 5.81 vs -4.20 ± 5.93, minimum -10.19 vs -15.46, maximum 6.38 vs 5.40, respectively. C) Lymph node metastasis negative vs positive, mean -1.81 ± 5.67 vs -5.67 ± 5.37, minimum -9.96 vs -15.46, maximum 6.38 vs 3.83, respectively. D) TNM clinical stage not advanced vs advanced: mean -2.34 ± 7.04 vs -4.43 ± 5.49, minimum -9.96 vs -15.46, maximum 6.38 vs 5.40, respectively.

The difference in gene expression of E-cadherin between OSCC cases (-4.3 ± 5.6) and controls (-0.3 ± 4.7) was significant (*p*< 0.001) (Fig. 1A), showing that E-cadherin was downregulated at the mRNA level in OSCC. Neither tumor size, lymph node status nor TNM stage showed a significant correlation with the E-cadherin mRNA level (Fig. 1B,C,D). However, we noted that patients with negative lymph node metastasis had a higher level of E-cadherin mRNA than that of patients with positive lymph node metastasis (-1.8 ± 5.6 vs -5.6 ± 5.3, respectively). Spearman’s rho was calculated with the aim of correlating the levels of mRNA E-cadherin with clinical parameters. Although the correlation was significant for all parameters evaluated, the rho value indicated a positive weak correlation for tumor size (*p* = .003 rho = .332) and invasion of lymph nodes (*p* = .019 rho = .268) but, strikingky, the correlation becomes weakly negative for the global clinical stage (*p* = .001 rho = -.389), where the trend line shows that as the clinical stage increases, the mRNA levels decrease.

**Figure 2:**
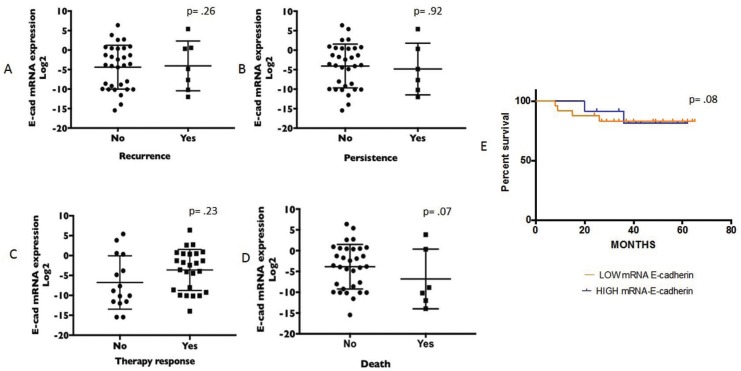
Comparison of the E-cadherin gene expression values and prospective data. A) Recurrence, no vs yes: mean -1.81 ± 5.32 vs -4.05 ± 6.38, minimum -13.00 vs -12.00, maximum 6.7 vs 5.4, respectively. B) Persistence, no vs yes: mean -4.19 ±5.30 vs -4.16 ± 7.16, minimum -13.95 vs -12.00, maximum 6.38 vs 5.40. C) Therapy response, no vs yes: mean -4.49 ± 6.53 vs -3.60 ± 5.15, minimum -12.00 vs -13.95, maximum 5.40 vs 6.38, respectively. D) Death, no vs yes: mean -3.79 ± 5.36 vs -6.80 ± 7.18, minimum -15.46 vs -13.95, maximum 6.38 vs 3.83, respectively. E) Kaplan–Meier survival curve for overall survival according to high/low E-cadherin mRNA expression and survival time. Survival with low E-cadherin mRNA expression (orange line) decreases earlier than with high E-cadherin mRNA expression (blue line).

Prospective data (recurrence, persistence, response to therapy and death) were also not significantly related to E-cadherin mRNA levels (Fig. 2A-D), although there was a trend for lower values of E-cadherin expression in deceased (-6.8 ± 7.1) vs. living patients (-3.79 ± 5.36).

The quantitative expression of E-cadherin was dichotomized in high and low expression groups using a value of 0 as the cutoff and was used to generate survival analysis curves. The average survival in the high expression group was 48.7 ± 3.3 months, and survival was reduced to 44.8 ± 3.3 months in the low expression group, although this difference was not significant. However, it can be seen in the survival curves that deaths occurred earlier in patients with low expression of E-cadherin (Fig. 2E).

**Figure 3:**
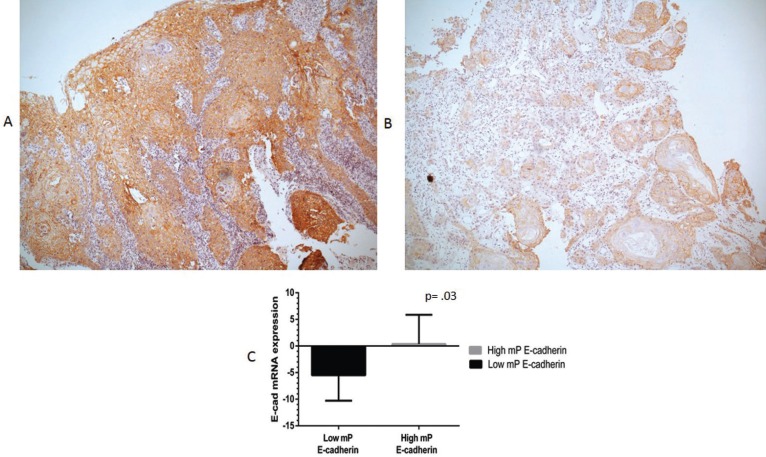
All tumors showed membranal protein (mP) E-cadherin expression at different intensities A) High mP expression of E-cadherin by immunohistochemistry in well-differentiated OSCC B) Low mP expression of E-cadherin by IHC in OSCC (magnification 40x). Mann-Whitney tests. C) Tissue with low mP E-cadherin: mean of -5.48 ± 4.8, minimum -12.00, maximum .92. Tissue with high mP E-cadherin: mean .39 ± 5.47, minimum -10.19, maximum 6.38.

E-cadherin at the cell membrane (mP E-cadherin) was marked by immunohistochemistry (Fig. 3A,B). Using Mann Whitney U test, a statistically significant relation between mP E-cadherin and E-cadherin mRNA levels in OSCC patients was observed (*p*=.03), as tissues with low mP E-cadherin expression mostly had low levels of E-cadherin mRNA, and conversely tissues with high mP E-cadherin expressed high levels of E-cadherin mRNA (Fig. 3C). As expected, therefore, similarly to E-cadherin mRNA levels, using the Chi-square test we found that mP E-cadherin was not significantly associated with the clinical and prospective parameters T (*p*= .67), N (*p*= .12), TNM clinical stage (*p*= .26) and recurrence (*p*= .86), persistence (*p*= .85), therapy response (*p*= .48) and death (*p*= .52).

## Discussion

E-cadherin downregulation at the mRNA level occurs due to several mechanisms and plays a key role in the cell motility that is required for the invasion and metastasis cancer hallmark to occur (29).

Despite the crucial role of E-cadherin downregulation in cancer, only two previous studies, to our knowledge, have analyzed the mRNA expression of E-cadherin in OSCC tumors. Wang *et al.* found that the expression of E-cadherin mRNA in the invasive tumor front was lower than in the central region (18). Their study did not report a comparison of the mRNA expression of E-cadherin with the clinical and prospective data of oral cancer patients. Yao *et al.* (23) analyzed the mRNA levels of E-cadherin by qRT-PCR using the 2- ΔΔCt method in 20 samples of oral cancer, but they used normal tissue adjacent to the tumor as the control group. In their study, they reported significant differences between tumoral tissue and adjacent normal tissue, but they did not report associations between E-cadherin mRNA expression and clinical parameters of OSCC.

Our study is, to the best of our knowledge, the first to analyze the expression of E-cadherin mRNA in tumors from patients with OSCC and in a real control group, as well as the first study to correlate E-cadherin mRNA levels with clinical and prospective characteristics of the disease. We consider that the most reliable control tissue is one that comes from a completely healthy area from an individual without OSCC, instead of tissue from an area adjacent to the tumor, which can be exposed to the same microenvironment as the tumor itself. The tumor microenviroment plays key roles during the initiation of the carcinogenesis process (29), and therefore normal-looking tissue adjacent to the tumor may carry some of the alterations in genes and signalling networks operating in the neighboring cancer cells, making such tissues an unreliable control.

In this study, E-cadherin mRNA expression was markedly diminished in OSCC tumors, in agreement with previous data on E-cadherin protein levels (11-13). However, low E-cadherin expression at the mRNA level occurred in the early clinical stages and did not continue to decrease significantly in the advanced stages, so its potential usefulness for OSCC staging seems limited. Nevertheless, we observed a trend towards further decreases in E-cadherin mRNA levels with positive lymph node metastasis and a weak negative correlation with TNM clinical stage.

One limitation of this type of studies is, on the one hand that E-cadherin gene expression, although constitutive, can be regulated by causes other than the carcinogenesis process, such as wound repair, inflammatory processes and immune response. On the other hand, there might be variations in the proportion of epithelial or mesenchymal tissue present in the tissue extracted from each sample, as well as variations in the precise tumor area from which the sample is obtained (front of tumoral advance or central tumor area), which constitutes another possible limitation of these studies.

Future studies with larger sample sizes will be needed to ascertain whether the E-cadherin mRNA level could eventually constitute a predictor of lymph node metastasis in OSCC. Such studies would also allow us to confirm our results regarding the non-significant correlations of E-cadherin mRNA levels with prospective variables such as persistence, recurrence and therapy response.

E-cadherin is regulated by several transcriptional factors that have emerged as important transcriptional repressors of E-cadherin during EMT in gastrulation, in addition to having specific roles in the tumorigenicity and metastatic behavior of squamous cell carcinomas *in vitro*. These E-cadherin regulatory mechanisms include the SNAIL superfamily (Snail 1 and Snail 2) (30), SIP1 (31), the microRNA family that includes miR200 and miR-2056,(32), proteins produced by the human papilloma virus type 16, E6 and E7 (the reduction of E-cadherin on the cell surface in the presence of these proteins has been shown *in vitro*) (33-35), as well as the coexpression of ErbB-2 (36).

Likewise, gene expression is also regulated epigenetically, regulating gene expression through changes in the structure of chromatin that results from the transfer of methyl groups.

Several authors have chosen to study concomitantly the expression of E-cadherin with some of the regulatory proteins in situ (22,37); thus, it would be interesting for future studies to include an evaluation of various transcriptional or regulatory factors and the epigenetics involved in the expression of E-cadherin specifically in oral squamous cell carcinomas.

In conclusion, our study confirms the importance of gene expression E-cadherin levels in OSCC and shows that its downregulation at the mRNA level is likely to be an early and constant event in the evolution of this disease. Further studies will be necessary to establish whether E-cadherin levels continue to decrease significantly as OSCC progresses.

## References

[1] Bray F (2014). Transitions in human development and the global cancer burden. In: Wild CP, Stewart B (ed). World cancer report 2014. Lyon: International Agency for Research on Cancer.

[2] Ferlay J, Soerjomataram I, Dikshit R, Eser S, Mathers C, Rebelo M (2012). Cancer incidence and mortality worldwide: Sources, methods and major patterns in GLOBOCAN 2012. Int J Cancer.

[3] Fernandes A, Pinto F, Shien L, Vamondes M, Cernea C (2015). Oral cavity squamous cell carcinoma: factors related to occult lymph node metastasis. Brazilian Journal of Otorhinolaryngology.

[4] Brennan M, Migliorati CA, Lockhart PB, Wray D, Al-Hashimi I, Axéll T (2007). Management of oral epithelial displasia: a review. Oral Surg Oral Med Oral Pathol Oral Radiol Endod.

[5] Massano J, Regateiro FS, Januario G, Ferreira A (2006). Oral squamous cell carcinoma: review of prognostic and predictive factors. Oral Surg Oral Med Oral Patho Oral Radiol Endod.

[6] Berx G, van Roy F (2009). Involvement of Members of the Cadherin Superfamily in Cancer. Cold Spring HarbPerspectBiol.

[7] Lau MT, Klausen C, Leung PCK (2011). E-cadherin inhibits tumor cell growth by suppressing PI3K/Akt signaling via β-catenin-Egr1-mediated PTEN expression. Oncogene.

[8] Barrallo-Gimeno A, Nieto MA (2005). The Snail genes as inducers of cell movement and survival: implications in development and cancer. Development.

[9] Caberg JH, Hubert PM, Begon DY, Herfs MF, Roncarati PJ, Boniver JJ (2008). Silencing of E7 oncogene restores functional E-cadherin expression in human papillomavirus 16-transformed keratinocytes. Carcinogenesis.

[10] Cobaleda C, Perez-Caro M, Vicente-Dueñas C, Sánchez-García I (2007). Function of the zinc-finger transcription factor SNAI2 in cáncer and development. Annu Rev Genet.

[11] Mattijssen V, Peters H, Schalkwijk L, Manni JJ, vant't Hof-Grootenboer B, de Mulder PH (1993). E-cadherin expression in head and neck quamous cell carcinomas is associated with clinical outcome. International Journal of Cancer.

[12] Diniz-Freitas Márcio, García-Caballero Tomás, Antúnez-López José, Gándara-Rey José Manuel, García-García Abel (2006). Reduced E-cadherin expresión is an indicator of unfavourable prognosis in oral squamous cell carcinoma. Oral Oncology.

[13] Zhao Z, Ge J, Sun Y, Tian L, Lu J, Liu M (2012). Is E-cadherina inmunoexpression a prognostic factor for head and neck squamous cell carcinoma (HNSCC)? A systematic review and meta-analysis. Oral Oncology.

[14] Jia-Yo W, Chen Y, Ho-Ren C, Duen-Jeng W, Wen-Chien C, Sheng-Yang L (2010). Potential biomarkers in saliva for oral squamous cell carcinoma. Oral Oncology.

[15] Yuwanati MB, Tupkari JV, Avadhani A (2011). Expression of E-cadherin in oral epithelial dysplasia and oral squamous cell carcinoma: An in vivo study. Journal of Clinical and Experimental Investigations.

[16] Andrews NA, Jones AS, Helliwell TR, Kinsella AR (1997). Expression of the E-cadherin-catenin cell adhesion complex in primary squamous cell carcinomas of the head and neck and their nodal metastases. Britishh Journal of Cancer.

[17] Tanaka N, Odajma T, Ogi K, Ikeda T, and Satoh M (2003). Expression of E-cadherin, α-catenin and β-catenin in the process of lymph node metastasis in oral squamous cell carcinoma. British Journal of Cancer.

[18] Wang X, Zhang J, Mingwen F, Zhou Q, Deng H, Aisharif MJ (2009). The expression of E-cadherin at the invasive tumor front of oral suqamous cell carcinoma: inmunohistochemical and RT-PCR analysis with clinicopathological correlation. Oral Surg Oral Med Oral Path Oral RadiolEndod.

[19] Rosado P, Leuqerica-Fernández P, Fernández S, Allonca E, Villallaín L, de Vicente JC (2013). E-cadherin and β-catenin expression in well-differentiated and moderately differentiated oral squamous cell carcinoma: relation with clinical variables. Br J Oral MaxillofacialSurg.

[20] Sasaya K, Sudo H, Maeda G, Kawashiri S and Imai K (2013). Concomitant Loss of p120-Catenina and β-catenin Membrane Expression and Oral Carcinoma Progression with E-cadherin Reduction. PLOS ONE.

[21] Pectasides E, Rampias T, Sasaki C, Perisanidis C, Kouloulias V, Burtness B (2014). Markers of Epithelial to Mesenchymal Transition in Association with Survival in Head and Neck Squamous Cell Carcinoma (HNSCC). PLOS One.

[22] Jiang Y, Liao L, Shrestha C, Ji S, Chen Y, Peng J (2015). Reduced expression of E-cadherin and p120-catenin and elevated expression of PLC- γ1 and PIKE are associated with aggressiveness of oral squamoous cell carcinoma. Int J Clin Exp Pathol.

[23] Yao X, Sun S, Zhou X, Zhang Q, Guo W, Zhang L (2017). Clinicopathological significance of ZEB-I and E-cadherin proteins in patients qith oral cavity squamous cell carcinoma. OncoTargets and Therapy.

[24] Meyerson M, Gabriel S, Gets G (2010). (Advances in understanding cáncer genomes through second-generation sequencing. Nature Reviews Genetics.

[25] (2010). AJCC: Lip and Oral cavity. En: Edge SB, Byrd DR, Compton CC, et al., 7th eds.

[26] (2016). National Cancer Institute.

[27] DeSouza M, Weslley P, Calsolari M (2015). Persistent and recurrent disease in ptient with papillary thyroid carcinoma with clinically apparent (cN1), but no extensive, lymph node involvement and without other factors for por prognosis. Arch Endocrinol Metab.

[28] Greene F, Page D, Fleming I (2003). AJCC Cancer Staging Hadbook Colon and rectum.

[29] Hanahan D, Weinberg R (2011). Hallmarks of cancer. The next generation. Cell.

[30] Olmeda D, Montes A, Moreno-Bueno G, Flores JM, Portillo F, Cano A (2008). Snai1 and Snai2 collaborate on tumor growth and metastasis properties of mouse skin carcinoma cell lines. Oncogene.

[31] Sobral LM, Sousa LO, Coletta RD, Cabral H, Greene LJ, Tajara EH (2014). Stable SET knockdown in head and neck squemous cell carcinoma promotes cell invasion and the mesenchymal-like phenotype in vitro, as well as necrosis, cisplatin sensitivity and lymph node metastasis in xenograft tumor models. Molecular Cancer.

[32] Tamagawa S, Beder LB, Hotomi M, Gunduz M, Yata K, Grenmar R (2014). Role of miR-200c/miR-141 in the regulation of epithelial-mesenchymal transition and migration in head and neck squamous cell carcinoma. Int J MoL Med.

[33] Carver EA, Jiang R, Lan Y, Oram KF, Gridley T (2001). The mouse snail gene encodes a key regulator of the epithelial mesenchymal transition. Mol Cell Biol.

[34] Subbaiah VK, Kranjec C, Thomas M, Banks L (2011). PDZ domains: the building blocks regulating tumorigenesis. Biochem J.

[35] D'Costa ZJ, Leong CM, Shields J, Matthews C, Hibma MH (2012). Screening of drugs to counteract human papillomavirus 16 E6 repression of E-cadherinexpression. Invest New Drugs. PLOS one.

[36] Al Moustafa AE, D Foulkes W, Benliname N, Wong A, Yen L, Bergeron J (2004). E6/E7 proteins of HPV type 16 and ErbB-2 cooperate to induce neoplastic transformation of primary normal oral epithelial cells. Oncogene.

[37] Wang C, Liu X, Chen Z, Huang H, Jin Y, Kolokythas A (2013). Polycomb group protein EZH2-mediated E-cadherin repression promotes metastasis of oral tongue squamous cell carcinoma. MolCarcinog.

